# Linguistic influence on mathematical development is specific rather than pervasive: revisiting the Chinese Number Advantage in Chinese and English children

**DOI:** 10.3389/fpsyg.2015.00203

**Published:** 2015-02-26

**Authors:** Winifred Mark, Ann Dowker

**Affiliations:** ^1^Department of Psychology, University of Hong KongHong Kong, China; ^2^Department of Experimental Psychology, University of OxfordOxford, UK

**Keywords:** linguistic transparency, counting system, arithmetic, cross-cultural, Chinese Number Advantage

## Abstract

The relative linguistic transparency of the Asian counting system has been used to explain Asian students’ relative superiority in cross-cultural comparisons of mathematics achievement. To test the validity and extent of linguistic transparency in accounting for mathematical abilities, this study tested Chinese and British primary school children. Children in Hong Kong can learn mathematics using languages with both regular (Chinese) and irregular (English) counting systems, depending on their schools’ medium of instruction. This makes it possible to compare groups with varying levels of exposure to the regular and irregular number systems within the same educational system, curriculum, and cultural environment. The study included three groups of first/second graders and third/fourth graders with varying degrees of experience to the Chinese language and counting systems: no experience (UK; *n* = 49); spoke Chinese at home and learnt to count in English at school (HK-E; *n* = 43); spoke Chinese at home and learnt to count in Chinese at school (HK-C; *n* = 47). They were compared on counting, numerical abilities and place value representation. The present study also measured nonverbal reasoning, attitude toward mathematics, involvement of parents, and extra-curricular mathematics lessons to explore alternative explanations of children’s numeric ability. Results indicated that students in HK-C were better at counting backward and on the numeric skills test than those in HK-E, who were in turn better than the UK students. However, there was no statistical difference in counting forward, place value understanding, and a measure of arithmetic. Our findings add to existent literature suggesting that linguistic transparency does not have an all-pervasive influence on cross-national differences in arithmetic performance.

## INTRODUCTION

International comparisons of children’s arithmetic performance, such as the Trends in International Mathematics and Science Study, consistently showed that Asian students outperformed their Western counterparts (Stedman, 1997; Mullis et al., 2000, 2008; Provasnik et al., 2012). While many individual and sociological factors could influence mathematics learning, the current study focused on linguistic influences on early mathematics learning. Recent years have seen a surge in empirical literature on the role of language in accounting for cross-cultural disparities in children’s number understanding and arithmetic competence (Fuson and Kwon, 1992; Aunio et al., 2004, 2006, 2008; Cheng and Chan, 2005; Rasmussen et al., 2006; Wang et al., 2008; Göbel et al., 2011; Krinzinger et al., 2011; Pixner et al., 2011; Zhao and Singh, 2011; Klein et al., 2013; Cankaya et al., 2014). Linguistic influences on mathematics learning warrant interest because the capacity to name and manipulate numeric quantities has been used to explain why human mathematical abilities could develop beyond the rudimentary number sense observed in animals (Dehaene, 1997). If the representation for large quantities and algorithms for calculation were underlay by language, it follows that distinct linguistic characteristics could lead to differential computational efficiency and arithmetic understanding.

There is a lot of debate about the extent to which language affects thought in general; but some evidence suggests that abstract concepts are more influenced than concrete ones by linguistic diversity (Gentner and Boroditsky, 2001; Borghi et al., 2011; Borghi and Binkofski, 2014). As number is a highly abstract concept, one might expect it to be more influenced by linguistic diversity than some other domains.

One linguistic characteristic that could influence children’s mathematics learning is the way in which numbers and arithmetical relationships are expressed in the counting system. It has been suggested that the superior arithmetic performance of Chinese and other Asian students could be explained by the relative *linguistic transparency* of many Asian counting systems (Fuson and Kwon, 1991; Miller et al., 2005; Ng and Rao, 2010), termed the ‘Chinese Number Advantage’ (CNA). Transparent number systems give a clear and consistent representation of the base system (base-ten in most languages). One example is the Chinese counting system, where the boundary between 10 and 11 is explicit in both written and spoken forms. The Chinese word for 11 is (*shi yi*), literally ‘ten–one’; that for 12 is (*shi er*), literally ‘ten–two,’ and so on. The same rule applies for larger numbers, such that 20 is (*er shi*) ‘two–ten,’ 59 is (*wu shi jiu*) ‘five–ten–nine’ and so on. Hence, new numbers could easily be inferred in Chinese, and it is clear that the numbers are organized according to a base-ten system.

Edgeworth and Edgeworth (1798) suggested more than 200 years ago that English-speakers might be at a disadvantage compared with speakers of other languages due to the relatively irregular English counting system. This gained empirical support from Miller et al. (1995), who found that Chinese and American 4- and 5- year olds performed similarly in learning to count up to 12, but the Chinese students were about a year ahead of the American children in the further development and counting of higher numbers. In contrast to regular counting systems, the English words *eleven* and *twelve* do not provide clear clues for their cardinality nor the base system. Those well-versed in the history of numbers might recognize that the English words for 11 and 12 reflected historical relations to the Old Saxon words *ellevan* and *twelif*, literally ‘one-left’ and ‘two-left’ respectively after 10 has been subtracted. However, this information is not apparent to young learners! In addition, various phonemic modifications further complicate number learning for English children: In 13–19, *ten* becomes -*teen*, *three* becomes *thir-*, and *five* becomes *fif-*. Above 19, *ten* becomes -*ty* for tens starting from 20, *two* becomes *twen-* in the twenties and *four* becomes *for-* in the forties.

English children also had more difficulties than speakers of some other languages in acquiring the base-ten system. Since English children must learn *one* through *twelve* by rote learning, the base-ten system might be scaffolded. Experimental evidence was provided by cross-cultural studies on six-year-olds using regular counting systems such as Chinese, Japanese, and Korean versus children from less regular counting systems such as French, Swedish, and the U. States. (Miura et al., 1988, 1993; Miura and Okamoto, 2003). Children were asked to represent numbers with cubes representing single units and ten-segmented blocks representing tens. It was found that children from regular counting systems were more likely to use bars and cubes in combination to represent numbers, while children from less regular counting systems were more likely to count out the exact number of cubes. Failure to take advantage of tens-bars suggested poorer understanding of the base-ten system.

The greater transparency of base system might make place value easier to grasp in a regular counting system (Miura and Okamoto, 2003). Place-value knowledge refers to the knowledge of the value of each digit by considering its place in a multi-digit number, such that each ‘5’ in 555 is understood as 5 hundreds, 5 tens, and 5 units, respectively. Such knowledge is essential for arithmetic computations. The regular Chinese number system can be directly mapped onto Arabic numbers; for example, 17 is ‘ten–seven’ in Chinese, making it obvious that the ‘1’ is a ’10.’ In contrast, place values of English numbers are obscured by the three forms of ten (*ten*, -*teen*, and -*ty*), and the fact that the order of reading numbers does not necessarily align with the Arabic numbers (e.g., *seventeen* vs. *seventy*). Such irregularities mask place values and hinder English children’s arithmetic development.

Despite the linguistic advantages that the Chinese number system potentially afforded, some considered that the CNA could not be an adequate explanation for Asian children’s superiority over Western children in nearly *all* mathematical domains (Ackerman, 1988). The many other cultural differences between Asian and Western children, such as quantity and quality of mathematics teaching (Saxton and Towse, 1998), attitudes of parents and personal motivation toward mathematics (Stevenson et al., 1993) weaken the CNA. Research conducted in Wales (MacLean and Whitburn, 1996; Dowker and Lloyd, 2005; Dowker et al., 2008) offered important insights in this regard, since groups with varied levels of exposure to regular (Welsh) and irregular (English) number systems could be compared. Dowker et al. (2008) found that Welsh children were facilitated on reading and comparing two-digit numbers, but not on all arithmetic tests. They concluded that linguistic transparency could not on its own explain the cross-national differences in arithmetic, thus providing indirect evidence against the CNA.

In a similar attempt to distinguish language and cultural effects, and to test the CNA directly, the present study recruited British and Hong Kong primary school students. The Hong Kong educational system is based upon the British system, reflecting its history as a British colony. Mathematics could be taught in a regular (Chinese) or irregular (English) counting system, depending on the medium of instruction of the school. It is hence possible to compare the mathematical performance of children who received either English- or Chinese-medium schooling, within the same educational system, curriculum, and cultural environment. Our study adds to the literature in that it is one of the first studies to take advantage of the Chinese/English medium of instruction system in Hong Kong to study linguistic influences in Mathematics. Our study also attempts to extend Dowker et al.’s (2008) Welsh study, as it also compares groups of children taught in different languages within otherwise similar settings. Furthermore, this study serves as a supplement to existing CNA studies, many of which compare Chinese and Finnish (Aunio et al., 2004, 2006, 2008).

Three groups of primary school children with varying degrees of experience with the Chinese language and counting system were compared in this study—those who had no experience (British students); those who spoke Chinese at home but learnt Mathematics in English (students in English-medium schools in Hong Kong); and those who spoke and learnt Mathematics in Chinese at both home and school (students in Chinese-medium schools in Hong Kong). The English- and Chinese-medium schoolchildren in Hong Kong differed mainly in terms of the linguistic medium used in their school instruction, but otherwise had similar cultural and educational experiences; while the British children were of course growing up within a different culture and educational system. They were all given a non-verbal intelligence measure, a test of numerical skills, a test of place value representations, and an attitude toward mathematics questionnaire. As both Chinese- and English-medium schools in Hong Kong followed the same mathematics curriculum, the two groups of Hong Kong children differed primarily in the language in which they learnt mathematics. Testing Hong Kong students taught in different media of instruction allowed us to tease apart whether it is the exposure to the Chinese language *per se* or the use of the Chinese counting system that influenced mathematical ability. British students served as a control group for exposure to the Chinese language, while students in the English-medium school in Hong Kong served as the control group for formal instruction of the Chinese counting system. The present study also took into account the role of children’s attitude toward mathematics and involvement of parents, both of which were often omitted in previous cross-linguistic studies (MacLean and Whitburn, 1996; Dowker et al., 2008).

Based on the CNA, it was hypothesized that (1) students in Hong Kong would perform better than British students on all numerical tasks, including counting, place value knowledge and the numerical skills test; (2) within Hong Kong, students in Hong Kong Chinese-medium schools would perform better than those in English-medium schools. In order to study the impact of duration in use of Chinese number system in numerical skills, we recruited a younger group (first-/second-graders) and an older group of children (third-/fourth-graders).

## MATERIALS AND METHODS

### PARTICIPANTS

A total of 159 children from two primary schools in Hong Kong and two primary schools in Oxford, UK participated in the testing. As the proximity of primary schools to students’ homes constitutes a major factor in primary school enrollment, the socioeconomic status (SES) of the catchment area in which the schools were situated could be considered proxy to the SES of their students. In this regard, the schools in Hong Kong and UK were located in predominantly middle-class areas. Testing in Hong Kong was done in August while testing in the UK was done in October of the same year. To ensure similarity of age and years in school, Hong Kong students were at the end of their first and third grade while UK students were at the start of their second and fourth grade. Written informed consent was obtained from parents of all participants. The study was approved by the Central University Research Ethics Committee of University of Oxford.

At the Chinese-medium school in Hong Kong (henceforth HK-C), Cantonese was the first language for all children, who came from Chinese-speaking homes. They received a Chinese-medium education, and were taught Mathematics in Cantonese. There were 25 first-graders and 25 third-graders from HK-C. At the English-medium school in Hong Kong (henceforth HK-E), Cantonese was the first language of the children, and they spoke Chinese at home. However, they received education in English for most school subjects including Mathematics. There were 37 first-graders and 16 third-graders from HK-E. At the British school in Oxford (henceforth UK), English was the first language of the children. They spoke English at home and at school, with no exposure to the Chinese language. There were 26 second-graders and 30 fourth-graders from UK.

### MEASURES

Measures employed were translated and back-translated from the English-version into Chinese by the first author and a bilingual experienced mathematics teacher, respectively. Two experienced mathematics teachers at a Chinese-medium primary school then reviewed all items.

#### Demographic and background information

Participants were asked about their age, grade and whether they attended kindergarten. To investigate the effect of additional mathematical instruction and parental involvement, participants were asked whether they attended mathematical classes outside of school and whether their parents helped them with their homework in general, as well as in math homework in particular.

#### Counting

Participants counted aloud from 1 to 30 and then backward from 30 to 1. Hesitations (more than 3 s delay), missing numbers, and incorrect sequence were recorded.

#### Numerical abilities

All children completed the British Abilities Scales (BAS) Basic Number Skills test, which involved recognizing and reading two-/three-digit numbers, as well as solving simple written calculations. Scores in *addition*, *subtraction*, *multiplication*, *division*, *fraction,* and *decimals* were added to compute a ‘purer’ measure of arithmetics. Raw scores were used in preference to standard scores as the test had not been standardized in Hong Kong.

#### Place value knowledge

Participants completed a number-comparison task identical to that used by Dowker et al. (2008), based on that of Donlan and Gourlay (1999). A pair of two-digit numbers was simultaneously presented to participants, who were asked to read them aloud and to point to the larger one within the pair. There were 24 pairs of numbers consisting of three types of number pairs: Transparent, Misleading, and Reversible. *Transparent* word pairs contained two numbers differing in the tens digit, thus requiring decade comparisons (e.g., 73 and 43) or contained repeated digits (e.g., 66 and 55). In *Misleading* number pairs, the smaller number contained a digit larger than the sum of digits in the larger item, (e.g., 51 and 47). *Reversible* pairs contained numbers whose tens and digit places were opposites (e.g., 85 and 58). An overall error score was calculated as in Dowker et al. (2008).

#### Attitude toward mathematics (ATM)

Mathematics and Anxiety Questionnaire (MAQ; Thomas and Dowker, 2000) was used to measure children’s ATM. Children answered four types of questions measuring self-perceived performance, attitudes in mathematics, unhappiness related to problems in mathematics, and anxiety related to problems in mathematics. There was a practice task followed by seven math-related situations: math in general, written calculations, mental calculations, easy calculations, difficult calculations, math homework, and listening and understanding the teacher during math lessons. Children answered on a 5-point scale using different pictures for each type of questions, such as ticks and crosses (“very good” to “very bad”), sweets and wasps (“like very much” to “hate very much”). The ratings varied from 0 for the most negative answer to 4 for the most positive answer, with a higher score indicating a more positive ATM. Overall the scale was found to be reliable (28 items, α = 0.89).

#### Non-verbal intelligence

All children completed Raven’s Colored Progressive Matrices Set A, AB, and B (Raven, 1962). Children were required to choose the correct answer from six options for 36 colored puzzles. Raven’s tests are favored as a measure of nonverbal intelligence since they are considered “culture-fair,” which is particularly important for cross-cultural studies. Raw scores were used in preference to standard scores as the available version of the test had not been standardized in Hong Kong.

## RESULTS

### DEMOGRAPHIC DATA

The means and SD of age, BAS total and arithmetic scores, Raven’s matrices, MAQ, and Number Comparison total error scores of the different Schools (language groups) are shown in **Table 1** and **2**. The variables were normally distributed, allowing subsequent parametric analyses. Participants with a Raven’s score two SD away from the group mean were excluded.

**Table 1:** Mean age, BAS total score, and arithmetic scores, Raven’s Matrices score, MAQ score, and Number Comparison task total error score for first/second grade students (SD in brackets).

Schools	N	Age	BAS		Raven	MAQ	Number comparison
			Total	Arithmetic			
HK-C	22	6.95 (0.21)	18.09 (5.41)	11.95 (4.04)	29.95 (3.00)	49.68 (10.10)	0.27 (0.88)
HK-E	27	6.65 (0.48)	16.96 (4.10)	11.81 (3.97)	29.74 (2.77)	67.67 (24.99)	1.44 (1.58)
UK	24	6.85 (0.29)	9.21 (3.38)	4.33 (2.33)	24.17 (3.84)	71.04 (14.28)	3.08 (3.88)

**Table 2:** Mean age, BAS total score, and arithmetic scores, Raven’s Matrices score, MAQ score, and Number Comparison task total error score for third/fourth grade students (SD in brackets).

Schools	N	Age	BAS		Raven	MAQ	Number comparison
			Total	Arithmetic			
HK-C	25	8.96 (0.35)	26.92 (2.83)	22.24 (3.96)	32.04 (3.13)	46.84(3.67)	1.36 (9.82)
HK-E	16	8.56 (0.51)	23.62 (3.85)	18.69 (3.00)	30.38 (3.24)	58.56 (12.54)	1.75 (2.82)
UK	25	8.88 (0.31)	16.28 (2.70)	10.22 (2.58)	26.32 (4.44)	64.76 (13.61)	0.48 (0.96)

### NONVERBAL INTELLIGENCE

Univariate ANOVA with School (three levels: HK-C, HK-E, UK) and Grade (two levels: first/second grade, third/fourth grade) as the independent variables (IV), and Raven’s score as the dependent variable (DV) was conducted to investigate whether students differed in intellectual functioning. Children at the three schools differed significantly on Raven’s matrices score, *F*(2,138) = 37.81, *p* < 0.001, η^2^ = 0.36. *Post hoc* LSD tests revealed that the difference was driven by the UK school and the Hong Kong schools (*p* < 0.001). The UK students had a lower score (μ = 25.27) than HK-E students (μ = 29.98) and HK-C students (μ = 31.06), while the Hong Kong schools did not differ significantly from each other. Children in the two grades were also significantly different from each other: *F*(1,138) = 7.43, *p* = 0.007, η^2^ = 0.053. Third/fourth grade students performed better than first/second grade students. There was no interaction of grade and school. Group differences in nonverbal intelligence were statistically controlled in subsequent analysis.

### COUNTING

**FIGURE 1:**
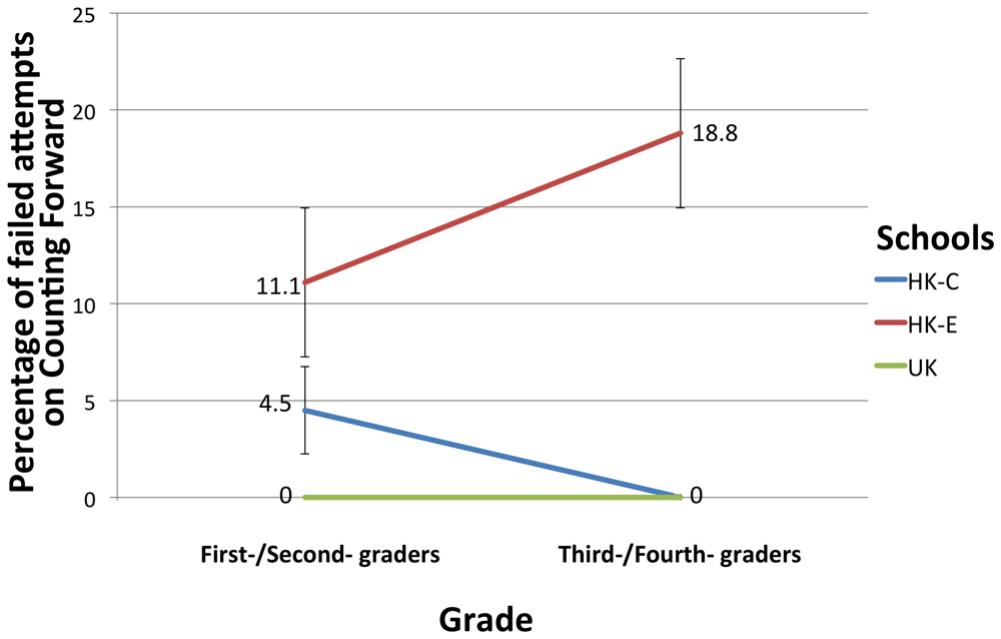
**Percentage of failed attempts at Counting Forward for students in the different Schools.** Error bars denote SEM.

**FIGURE 2:**
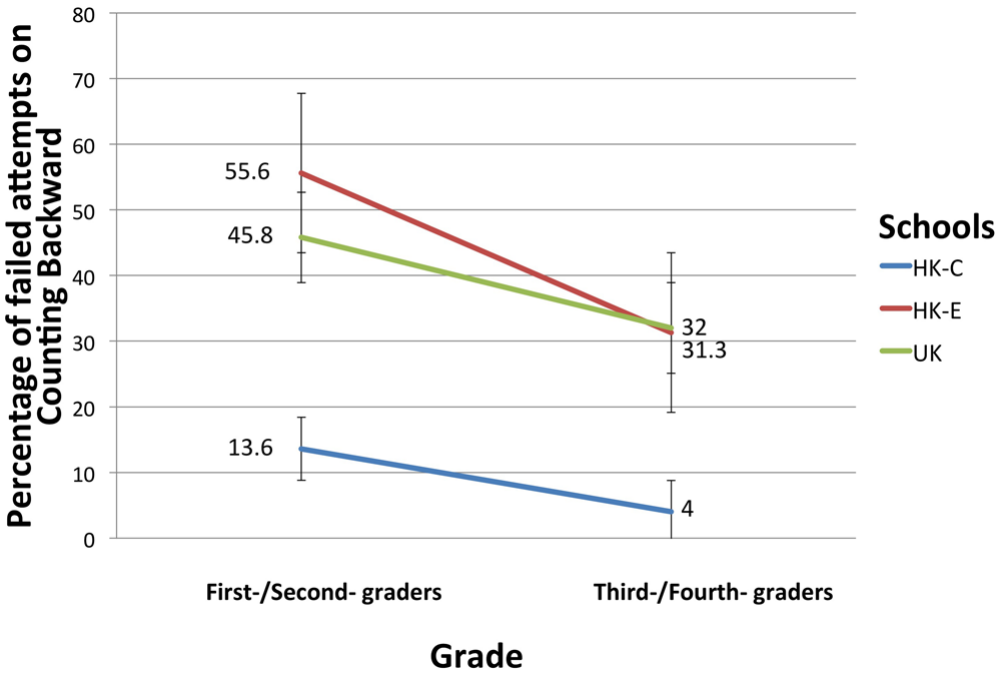
**Percentage of failed attempts at Counting Backward for students in the different Schools.** Error bars denote SEM.

For the Counting task, a successful attempt was one in which no mistakes were made. Hesitations of over three seconds, incorrect sequence or missing numbers constituted a failed attempt. Chi-squared contingency tests revealed a non-significant relationship between Schools and Success/Failure on the Counting Forward task for first-/second-graders. However, there was a significant relationship between Schools and Success/Failure on the task for third-/fourth-graders, *X*^2^(2, *N* = 66) = 9.82, *p* = 0.007. **Figure 1** depicted percentage of students who failed the Counting Forward task. Chi-squared test results for Counting Backward tasks revealed a significant relationship between Schools and Success/Failure on the task for first-/second-graders, *X*^2^(2, *N* = 73) = 9.45, *p* = 0.009. There was also a significant relationship between Schools and Success/Failure for third-/fourth-graders, *X*^2^(2, *N* = 66) = 7.14, *p* = 0.028. In both Counting Forward and Backward tasks, paired comparisons between groups were not possible due to relatively small sample sizes. **Figure 2** depicted percentage of students who failed the Counting Backward task.

### NUMBER COMPARISON

**FIGURE 3:**
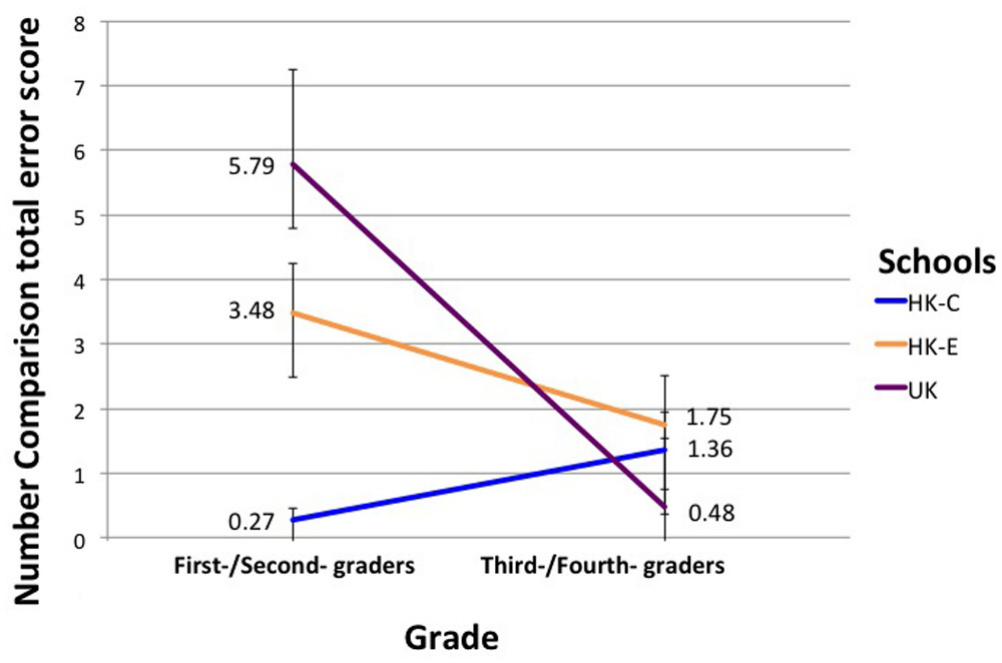
**Interaction effect of Grade and School on Number Comparison error scores.** Error bars denote SEM.

Univariate ANCOVA with Grade and School as IV, Number Comparison total error score as DV and Raven’s matrices score as a covariate showed that Grade *F*(2,132) = 7.92, *p* = 0.001, η^2^ = 0.11, as plotted in **Figure 3**. *Post hoc* pairwise comparisons showed that HK-C students were significantly better than HK-E and UK students in first/second grade: *F*(2,132) = 7.168, *p* = 0.001, η^2^ = 0.098, but not in third/fourth grade. Also, within the UK group, first-/second-graders had higher error scores than third-/fourth-graders: *F*(1,132) = 19.007, *p* < 0.001, η^2^ = 0.13.

### NUMERICAL SKILLS AND ATTITUDES

To investigate the effect of linguistic influences on numerical skills, a MANCOVA with Grade and School as IV, MAQ, and BAS total scores as DV and Raven’s matrices score as a covariate showed a significant effect of Grade and BAS total scores: *F*(1,132) = 118.54, *p* < 0.001, η^2^ = 0.47. Third/fourth grade students performed better on the BAS total scores than first/second grade students even after controlling for IQ. A significant effect of School and BAS total scores was found: *F*(2,132) = 41.98, *p* < 0.001, η^2^ = 0.39. *Post hoc* tests revealed that BAS total scores from all schools significantly differed from each other, even after controlling for IQ. HK-C students performed the best, followed by HK-E students and then UK students. There was also a significant effect of Grade and MAQ: *F*(1,132) = 5.10, *p* = 0.026, η^2^ = 0.04, with first/second grade students having a higher MAQ score. There was also a significant effect of School and MAQ: *F*(2,132) = 16.07, *p* < 0.001, η^2^ = 0.20. *Post hoc* tests revealed that HK-C students scored lower than HK-E students and UK students, but HK-E students did not differ significantly from UK students. There was no significant interaction between Grade and School, for either BAS total score or MAQ.

**FIGURE 4:**
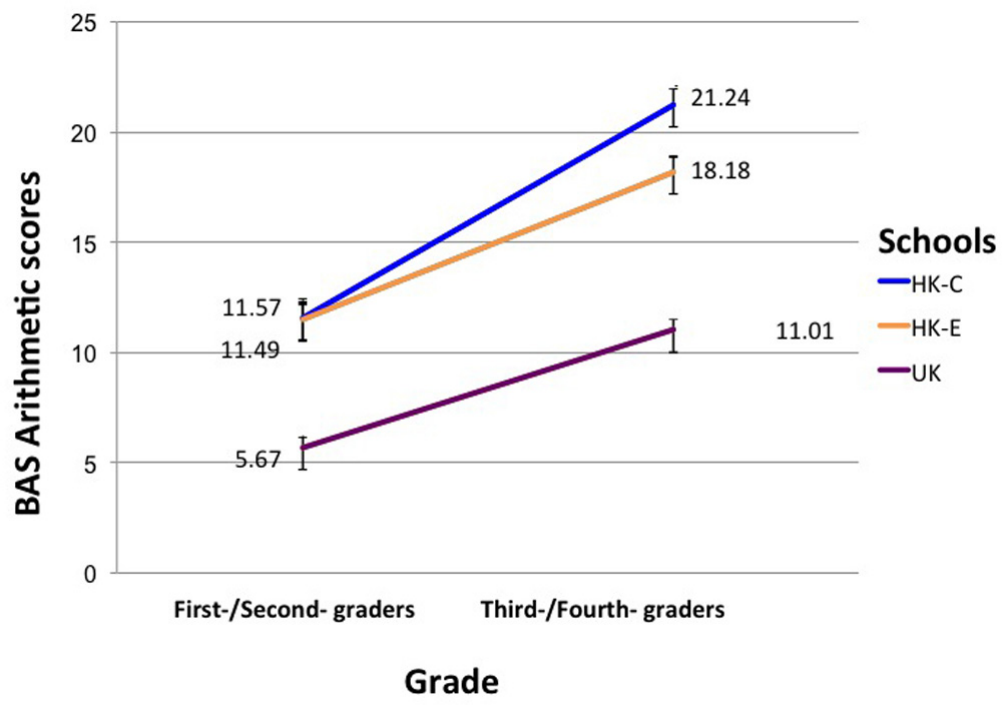
**Interaction effect of Grade and School on BAS arithmetic scores.** Error bars denote SEM.

To investigate linguistic influences on arithmetic abilities specifically, a MANCOVA with Grade and School as IV, MAQ, and BAS arithmetics as DV and Raven’s matrices score as a covariate was conducted. Results showed significant main effects of Grade and BAS arithmetic score: *F*(1,130) = 154.34, *p* < 0.001, η^2^ = 0.54, as well as School and BAS arithmetics: *F*(2,130) = 49.36, *p* < 0.001, η^2^ = 0.43. A significant interaction was found between Grade and School for BAS arithmetics: *F*(2,130) = 5.31, *p* = 0.006, η^2^ = 0.76. The interaction is plotted in **Figure 4**. First-/second-graders in the two Hong Kong schools did not significantly differ from each other in BAS arithmetic, but the UK first-/second-graders performed worse than the HK students. In third-/fourth-graders, all the schools differed in performance, with HK-C students performing better than HK-E students, who were in turn better than UK students in arithmetics.

To investigate whether group differences in MAQ might be either exaggerating or masking group differences in arithmetical performance, a final ANCOVA was carried out with Grade and School as IV, BAS arithmetic as DV, and both Ravens and MAQ as covariates. There was again a significant effect of both Grade [*F*(1,129) = 33.44; *p* < 0.026; η^2^ = 0.94] and of School [*F*(2,129) = 12.42; *p* < 0.059; η^2^ = 0.92], with a significant interaction between School and Grade: *F*(2,129) = 5.01; *p* = 0.008, η^2^ = 0.072. Ravens continued to be a significant covariate of Grade [*F*(1,129) = 12.85; *p* < 0.001; η^2^ = 0.091] but MAQ was not.

### PARENTAL INVOLVEMENT AND OUTSIDE SCHOOL MATHEMATICS

In order to establish whether parental involvement had an influence on the students’ performance on BAS in the different schools, a chi-squared contingency test was conducted between Parental Involvement and Schools for first-/second-graders and third-/fourth-graders separately. No significant results were found in either grade, indicating that parental involvement in math did not differ significantly across the schools.

Similarly, to establish whether outside school mathematics instruction might have an influence on the students’ performance in BAS in different schools, a chi-squared contingency test was carried out between Outside School Mathematics and Schools for first-/second-graders and third-/fourth-graders separately. No significant results were found in either grade. Therefore, the students’ outside school formal mathematics instruction in math did not differ significantly across the three schools.

## DISCUSSION

### GENERAL DISCUSSION

This study aimed at examining the effect of exposure to the transparent Chinese counting system on counting, place value understanding general mathematical performance, and arithmetics through a cross-cultural study of HK-C, HK-E, and UK children. Our experimental design allows us to posit that if children performed in a descending order of HK-C > HK-C > UK, formal instruction and use of Mathematics in Chinese might be driving the difference in performance. If Hong Kong schoolchildren performed as a group performed better than UK children, however, it could be suggested that mere exposure or knowledge of Chinese counting system was enough to impact performance. The effect of duration of use of a Chinese counting system was also studied through the comparison of younger (first/second grade) and older (third/fourth grade) children. Our results showed that CNA imposed an effect on general mathematical abilities (BAS total scores). However, this effect was not apparent in relation to number representation (Counting Forward/Backward), or arithmetic abilities (BAS arithmetic scores) specifically. Furthermore, exposure to Chinese counting systems was only found to impact place value knowledge (Number Comparison) in younger, but not older, children. This suggested that while exposure to a regular counting system could advance place value understanding and general numerical abilities, it is unlikely to confer long-term benefits nor be able to explain cross-national differences in arithmetic abilities specifically.

In an attempt to exclude confounding variables to examine linguistic influences, our study minimizes the effects of known alternative explanations to CNA in explaining number representations and arithmetic performance, such as IQ, ATM, outside school mathematics classes and parental involvement in children’s learning.

#### IQ

The UK students in our sample had a lower Raven’s score than the Hong Kong groups. Since nonverbal IQ is related to general academic abilities including arithmetics, this effect was controlled as a covariate. Given that the correlations and group comparisons all controlled for the effect of IQ, significant differences obtained were explicable by some factors over and beyond the influence of IQ.

#### Attitude toward mathematics

It has been suggested that more positive attitudes in Chinese compared to American students might contribute to the former putting more effort into their learning (Wong et al., 2001). However, our results showed that UK students indicated a more positive ATM, followed by HK-E and then HK-C students, which is the exact opposite of their pattern of performance on the arithmetic test. Our findings were in line with previous findings that students in countries ranking high in international comparisons disliked mathematics (Leung et al., 2006; Hirabayashi, 2006). Moreover, when attitude score was included as a covariate in the analysis of the effects of group and grade, it was found neither to affect BAS scores, nor to change the nature of the group differences. We cannot, however, exclude the possibility that Hong Kong students were more motivated to do well in academic assessments in general. It has previously been suggested that Chinese students were driven by pleasure derived from a result of the success attained in exams rather than through the process of learning *per se* (Leung et al., 2006).

#### Outside school mathematics classes

Some students enrolled in tutorial classes outside of school. These tutorial classes are not usually subject-specific for primary school students, though some are (e.g., the Kumon Educational maths program). Although it could be expected that exposure and drilling in arithmetics might impact positively on arithmetic tests, extra-curricular mathematics class participation did not differ significantly across the three schools in our study. Hence, outside school mathematics exposure is unlikely to be responsible for the cross-cultural differences in arithmetic abilities found in our study, though one cannot rule out possible influences of more specific characteristics of the extracurricular instruction provided to different children.

#### Parental involvement in children’s education

Some studies suggested that Chinese parents were more involved in their children’s education, giving more help or reprimand (Chen and Stevenson, 1989). Thus we asked students if their parents helped with their mathematics homework or taught them mathematics at home. However, our results showed that there was no statistically significant difference in self-reported level of parental involvement across the schools. Hence differences in arithmetic abilities found in this study were not likely to be due to disparate parental involvement, though it is always necessary to be cautious about self-report measures.

#### Curriculum and educational system

The primary educational system in Hong Kong is modeled on that of the British system, reflecting its colonial history to the UK. In Hong Kong, children receive primary education ‘Primary 1 – Primary 6’ from the ages of 6 until 12. In England, primary education spans over a similar age range, and is divided into ‘Key Stage 1’ (5–7 years old) and ‘Key Stage 2’ (7–11 years old). In both education systems, schools are required to teach a curriculum set by the government. The HK-C and HK-E students shared the same curriculum and Confucian traditions for academic excellence. In Hong Kong, more than half of primary school children were allocated centrally to Chinese-medium or English-medium schools. Hence, selection bias in relation to medium of instruction was unlikely to severely undermine our results. While the curriculum difference between Hong Kong and the UK was not possible to control, Tsang and Rowland (2005) had concluded that the two curricula were similar in content and organization. However, the scope of the study did not permit detailed comparisons of the implemented curricula and classroom teachings across the schools.

There are also other possible differences between the schools, which could have conceivably affected the results. Although there was no explicit difference in prestige or selectivity between the schools, and they were in similar neighborhoods, it is still possible that there might have been subtle differences between the parents, who chose to send their children to the Chinese- and English-medium schools. For example, the parents, who sent their children to the Chinese-medium school, might have identified more closely with Chinese culture, including an emphasis on mathematics and science. It may also be that some of the parents, who sent their children to the English-medium school, may have been responding to lower perceived mathematical ability in the children, by sending them to a school where they might compensate by acquiring fluency in a foreign language. The fact that the two Hong Kong groups did not differ in Ravens score reduces the likelihood that the differences in mathematical performance were due to some important pre-existing differences in ability; but one cannot rule out such differences altogether.

### COUNTING

Interestingly, UK students were found to be better than Hong Kong students at forward counting from 1 to 30 in first/second grade. This was inconsistent with the idea that regular number systems required less cognitive effort to learn and thus should be learnt earlier (Miller et al., 1995; Towse and Saxton, 1998). The observably poorer performance of HK-E students in forward counting across grades highlighted the caveat that it could not be determined whether the HK-E students could be counting or reading the numbers in Chinese in their heads and then giving an English response. Hence, their poor performance could be due to having to give response in a second language, especially one which is less transparent. It should be noted that it would be inevitably difficult to obtain a sample with no ‘contamination’ of a second language in any likely setting for a bilingual educational system.

In Backward Counting from 30 to 1, Hong Kong students appeared to perform better than their HK-E and UK peers, but the difference between HK-E and UK children was minimal. Taking together the results of Forward and Backward Counting, it could be suggested that Forward Counting consisted of rote learning of the sounds of number strings; hence it might not be a real indication of children’s number counting ability. When children were asked to count backward, which was much less common to hear and produce, the results showed a difference between children learning to count with a regular Chinese counting system and irregular English counting system. Since Chinese has a more transparent counting system, it is easy to infer the next number up or down the number line. Thus, students who learnt to count in Chinese could easily produce the backward sequence on the spot. In contrast, children who learnt to count in English had more difficulty, as it required ‘flipping over’ their phonological representation of the number strings.

### NUMBER COMPARISON

Our finding that HK-C students were significantly better than HK-E and UK students on the number comparison task in younger children but not older children suggested that transparency of the Chinese counting system might give children a ‘head-start’ in place value understanding. However, such an advantage bestowed by the CNA on first/second grade children was not ‘sustainable,’ as students who learn to count in irregular English counting system gradually ‘caught up’ in place value knowledge, as shown by the non-significant difference in the number comparison task across schools in third/fourth grade. That being said, such a conclusion is limited by a cross-sectional design and needs to be clarified in a longitudinal study in which children from HK-C, HK-E, and UK are followed through from first/second grade to third/fourth grade on the same task. It would also be interesting to replicate our study with an addition of a more explicit measure of place value knowledge (e.g., base-ten blocks) than our Number Comparison task. Our results support the practice of teaching young children from irregular counting systems to learn how numbers are formed in transparent number systems. Such an experience could serve both as cultural exposure and as a means to gain insight into the base system and place values.

### MATHEMATICAL COMPETENCE

Numerical competence was tested with the British Abilities Scale Number Skills Test, which was developed for students following the UK curriculum. Despite this potential advantage to the UK students, they performed the worst out of the three groups. Our results revealed an expected descending order of performance (HK-C, HK-E, UK) on general mathematic performance as measured by the total score on the BAS Number Skills test, as well as arithmetic performance in older children. Interestingly, however, such a disparity was not found for questions tapping arithmetic operations in younger children. HK-C did not achieve better arithmetic performance relative to HK-E children in the first/second grade. However, Hong Kong students as a whole still performed better than children in the UK.

As noted above, one caveat was that HK-E students could be disadvantaged by having to learn and respond in a second language. However, Dowker et al. (2008) showed that the advantages of learning Mathematics in Welsh held even if it was not a child’s first or only language. Hence, the poorer performance of HK-E relative to HK-C children was unlikely to be due to disadvantages of learning in a second language. Although our results could be interpreted to mean that some exposure to a regular Chinese system was still advantageous even if it was not the formal medium of instruction at school, our results weaken the CNA *per se* as an explanation for better arithmetic abilities of Asian students.

### CONCLUSION AND FUTURE STUDIES

In conclusion, this study demonstrated that young children who were learning mathematics in Chinese were better at manipulating the number line than those learning mathematics in English, whether English be their first or second language. We also showed that linguistic transparency in number representations might facilitate place value learning in young children, but such an advantage is neither sustainable nor necessarily translated to better arithmetic performance in older children. Our pattern of findings replicated that of Dowker et al. (2008), whereby children who learnt mathematics in regular counting system out-performed those who learnt mathematics in English on the Number Comparison task but not (at least for the younger children) on a test of more general arithmetic. The mechanism underlying the linguistic influence is, however, yet to be elucidated. As yet, the evidence is not sufficient to demonstrate that the CNA, as framed in terms of transparency of numbers, can explain the cross-national differences in arithmetic consistently demonstrated across age groups. The fact that children in HK-E performed significantly better than the UK children, although both groups were educated in English, suggests that general educational and cultural differences are at least as important as linguistic differences; though one cannot rule out the possibility that the HK-E children were advantaged by their exposure to Chinese counting at home.

More research is needed to fully understand the nature and extent of the differences in arithmetic between Chinese- and English-speaking children. To date, only a few studies have taken advantage of the unique opportunities afforded by the Chinese- and English-medium of instruction to tap linguistic influence in mathematics learning. In an ideal world, children of similar backgrounds would be randomly assigned to Chinese versus English medium schools, to rule out any effects of self-selection. In practice, this would of course be impossible. However, extending the number of schools studied would reduce the chances of the results being due to sample or school characteristics that are unrelated to language. It would also be interesting if the study could be extended to even younger children in kindergarten in order to test for even earlier effects. Moreover, it would be desirable to include a wider variety of number representation tasks: for example, including the blocks task of Miura et al. (1988). It is a potential limitation that the British Abilities Scales and the Raven’s Matrices were developed for use in Britain, rather than in Hong Kong. The fact that Hong Kong pupils outperformed British pupils on both tests makes it in fact unlikely that these tests involved unfamiliar or unsuitable material for use in Hong Kong schools. However, future studies should also attempt to develop and standardize tests for simultaneous use in the UK and in Hong Kong.

## Conflict of Interest Statement

The authors declare that the research was conducted in the absence of any commercial or financial relationships that could be construed as a potential conflict of interest.
