# Corrigendum: Linguistic influence on mathematical development is specific rather than pervasive: revisiting the Chinese Number Advantage in Chinese and English children

**DOI:** 10.3389/fpsyg.2016.00342

**Published:** 2016-03-16

**Authors:** Winifred Mark, Ann Dowker

**Affiliations:** ^1^Department of Psychology, University of Hong KongHong Kong, China; ^2^Department of Experimental Psychology, Oxford UniversityOxford, UK

**Keywords:** linguistic transparency, counting system, arithmetic, cross-cultural, Chinese Number Advantage

Due to an oversight, the two sentences preceding the final sentence in the abstract should be changed to read: Results indicated that students in HK-C were better at counting backward than those in HKE, who were in turn better than the UK students. However, there was no statistical difference in counting forward or place value understanding. Children in both Hong Kong schools performed better at the arithmetic test than the UK children. Among the older group, the HK-C children performed better on the arithmetic test than the HK-E children, but no such difference was found in the younger group.

The authors apologize for this mistake.

This error does not change the scientific conclusions of the article in any way.

## Author contributions

All authors listed, have made substantial, direct and intellectual contribution to the work, and approved it for publication.

### Conflict of interest statement

The authors declare that the research was conducted in the absence of any commercial or financial relationships that could be construed as a potential conflict of interest.

